# Accuracy of Mandibular Foramen Localization Using Digital Orthopantomogram (OPG) in Middle Eastern Population

**DOI:** 10.3390/diagnostics14192173

**Published:** 2024-09-29

**Authors:** Yasser S. Alali, Wajdi A. Mohammed (Bin), Sami M. Alotaibi, Sami Alshehri, Muath Alshayban

**Affiliations:** 1 Department of Oral and Maxillofacial Surgery, College of Dentistry, King Saud University, Riyadh 11545, Saudi Arabia; samalotaibi@ksu.edu.sa; 2 Department of Oral Medicine and Diagnostic Sciences, College of Dentistry, King Saud University, Riyadh 11545, Saudi Arabia; wbinmohammed@ksu.edu.sa; 3 Department of Biomedical Dental Sciences, College of Dentistry, Imam Abdulrahman Bin Faisal University, Dammam 31441, Saudi Arabia; smalshehri@iau.edu.sa; 4 Department of Restorative Dental Sciences, College of Dentistry, King Saud University, Riyadh 11545, Saudi Arabia; malshayban@ksu.edu.sa

**Keywords:** orthopantomogram, OPG, cone-beam computed tomography, CBCT, panoramic radiograph, digital imaging, mandibular foramen, anatomic localization, accuracy

## Abstract

Background/Objectives: Locating the mandibular foramen (MF) through imaging is clinically important for inferior alveolar nerve (IAN) anesthesia and mandibular ramus osteotomies. Although cone-beam computed tomography (CBCT) is superior in imaging the mandible, an orthopantomogram (OPG) is preferred for its ease of use and availability. Therefore, the present study aimed to evaluate the accuracy of digital OPG in localizing the MF, in a subset of the Middle Eastern population. Methods: Radiographic images (OPG and CBCT) of selected patients (adults, dentulous and no mandibular abnormalities) were used to locate the MF through digital measurements (mm) of the anteroposterior distance from the anterior border of the ramus (MF-AP) and the superoinferior position from the mandibular occlusal plane (MF-SI). Measurements were statistically compared between OPG and CBCT for accuracy. Differences in measurements between OPG and CBCT were compared against the anatomic location (right/left), age and biological sex, assuming a *p*-value < 0.05 as significant. Results: A total of 204 radiographic records (males: 100/females: 104/mean age: 34.65 ± 11.55 years) were evaluated. The measurements for the MF were MF-AP-OPG (right: 13.53 ± 2.44/left: 13.19 ± 2.25), MF-AP-CBCT (right: 13.61 ± 2.39/left: 13.36 ± 2.19), MF-SI-OPG (right: 5.25 ± 1.71/left: 5.41 ± 1.65) and MF-SI-CBCT (right: 5.59 ± 1.66/left: 5.52 ± 1.61). Measurements between OPG and CBCT were not significantly different, except for MF-SI (right) (*p* = 0.042). While the overall difference between OPG and CBCT (MF-AP/MF-SI) measurements showed a significant association (*p* < 0.01) with the anatomic location (right/left), a significant association (*p* < 0.05) with biological sex was observed only for MF-AP. Conclusions: Based on this study’s outcomes, digital OPG is an accurate modality to locate the MF based on anteroposterior (MF-AP) and superoinferior (MF-SI) measurements. This would be clinically beneficial for dental and oral surgeons to achieve the optimum IAN block anesthesia based on preoperative panoramic radiographs. Similarly, it would assist maxillofacial surgeons in planning mandibular orthognathic surgeries and ramus osteotomies without complications.

## 1. Introduction

**Figure 1:**
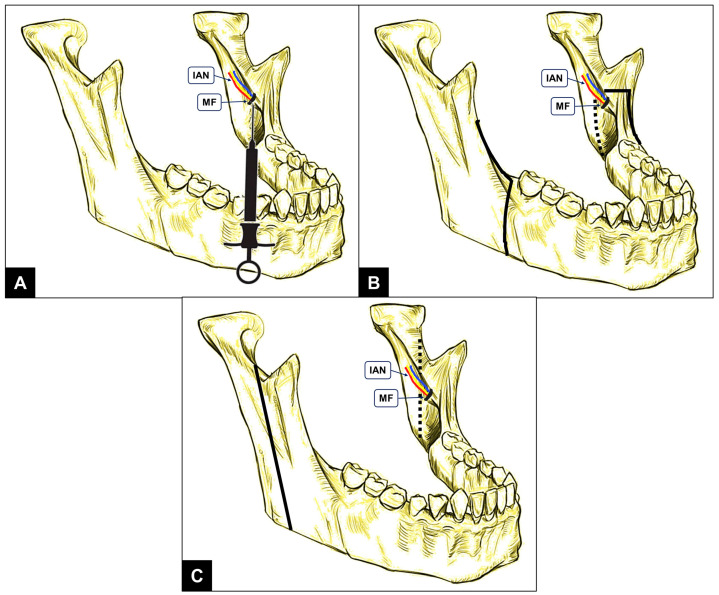
Representative line drawings of the mandible, showing the inferior alveolar neurovascular bundle (IAN) entering the mandibular foramen (MF) and the significance of the MF position in different clinical scenarios: (**A**) needle target site during administration of inferior alveolar nerve block; (**B**) orthognathic surgery bone cuts (solid and dotted black lines) for sagittal split mandibular osteotomy; and (**C**) vertical ramus osteotomy.

The mandibular foramen (MF) is an important anatomic structure in the medial aspect of the ramus of the mandible [1]. The inferior alveolar nerve (IAN), a branch of the posterior division of the mandibular nerve, enters the mandibular canal through the MF and innervates all of the mandibular teeth [2]. The mandibular canal or IAN canal houses the inferior alveolar/dental neurovascular bundle and extends from the MF, at its proximal end, to the mental foramen, at the distal end [2]. Immediately superior to the MF is a triangular projection of bone called the lingula [3]. Knowledge about the location of the MF is imperative for clinical practice in dentistry, oral surgery and maxillofacial surgery, as it relates to the success of IAN block anesthesia and the avoidance of complications during mandibular orthognathic surgeries [3,4]. The technique of IAN block requires clinicians to deposit a local anesthetic (LA) solution in the pterygomandibular space (bounded between the medial surface of the mandibular ramus and the medial pterygoid muscles) at a target site close to the MF (Figure 1A) [5]. Similarly, during mandibular orthognathic surgical procedures, such as sagittal split osteotomy and vertical ramus osteotomy, the bone cuts are created immediately posteriorly and in close proximity to the MF (Figure 1B,C) [6]. This is performed in order to retain the IAN as it enters the mandibular canal within the distal osteotomy segment [6]. Considering the aforementioned clinical circumstances, the radiographic localization of the MF is imperative to achieve successful local anesthetic delivery to the IAN, optimize the planning of mandibular orthognathic surgery and prevent iatrogenic injuries of the IAN [3,7,8]. However, mandibular anatomic variations due to factors such as age, sex, facial asymmetry and skeletal abnormalities are capable of altering the position of the MF between individuals [9].

Studies based on the clinical evaluation of patients without morphological abnormalities suggest the location of the MF to be slightly posterior to the midpoint in an anteroposterior orientation and at the level of the occlusal plane or slightly superior to it in a superoinferior direction [10,11]. Similarly, based on cadaveric dissection and observations of dry mandibles, the position of the MF is reported to be approximately 2.75 mm posterior to the midpoint of the ramus and about 19 mm inferior to the coronoid notch [12,13,14]. Nevertheless, these measurements pertaining to the location of the MF could vary between patients due to anatomic differences relating to age, biological sex, population and anthropological characteristics and within the same patient between the right and left sides [15]. A simple case in point is the relative superoinferior position of the MF with respect to the occlusal plane, which tends to be level in children and is more superiorly placed with advancing age [14]. Similar differences are observed in the anteroposterior position of the MF between males and females and between individuals with skeletal class III and class I/II, due to the large mandibular ramus in males and class III patients [16]. It is therefore important, from the perspective of a clinician administering IAN block anesthesia or a maxillofacial surgeon planning orthognathic surgery in the mandibular ramus, to appreciate the variations in the MF position and to ascertain it preoperatively using radiographs [3,17,18].

With the advancement of radiographic imaging techniques, the localization of the MF has become possible with replicable accuracy [4]. An orthopantomogram (OPG) is a panoramic radiograph of the maxilla, mandible, temporomandibular joints and teeth [14]. OPG is among the oldest reported radiographic modalities used for the localization of maxillary and mandibular anatomic structures and is commonly used for oral and maxillofacial surgical planning, owing to its simplicity, efficiency and ease of use [15,16]. In spite of their merits, panoramic radiographs are prone to inaccuracies due to the tomographic and two-dimensional nature of image acquisition [14]. However, in the last decade, digital image acquisition techniques have led to the replacement of OPG films with digital OPG images or panoramic radiographs [19]. Digital OPGs are not only time-efficient but also enable direct linear measurements between anatomic landmarks [19]. Based on a study comparing measurements on panoramic radiographs of dry skulls and direct measurements with calipers, Patil et al. [12] reported OPG as a reliable radiographic technique for the localization of mandibular anatomic structures, including the MF [12]. Similarly, Neves et al. [15] reported the considerable efficacy of OPG in accurately locating the mandibular anatomic landmarks and variations therein [15].

On the other hand, cone-beam computed tomography (CBCT) is a portable variation of computed axial tomography (CAT) for the digital, three-dimensional (3D) imaging of orofacial structures [4]. In addition to providing accurate images of maxillomandibular anatomic structures, CBCT is also capable of 3D volumetric reconstruction and software-based panoramic image rendering and measurements [6,20]. Several studies in the literature have reported the relative ease and accuracy with which CBCT can be used to identify mandibular anatomical structures along with their variations, including the MF, mandibular canal, lingula, mental foramen and accessory foramina [4,5,6,21,22,23,24]. Although CBCT is largely replacing OPG and digital panoramic radiographs in all clinical dental scenarios, it is mostly available in large clinical centers and healthcare institutions, owing to the high cost and the complexity of the setup and maintenance [15]. Meanwhile, conventional panoramic imaging using digital OPG is a relatively simpler radiographic technique, being available in small and large dental healthcare institutions alike [12]. In light of the aforementioned contradictions associated with OPG and CBCT, it is possible to state that digital OPG is an accurate alternative to CBCT for the localization of maxillomandibular anatomic structures, including the MF [12,15].

Considering the clinical importance of identifying and locating the MF before dental, oral and maxillofacial surgical procedures, the present study sought to evaluate the accuracy of digital OPG for this purpose. Therefore, the aim of the present study was to evaluate the accuracy of localizing the MF in the anteroposterior and superoinferior directions using panoramic radiographic images obtained through digital OPG, in a subset of the Middle Eastern population. In order to perform a comparison, panoramic radiographic images obtained through CBCT imaging were used as a benchmark.

## 2. Materials and Methods

### 2.1. Ethical Approval, Study Design and Sampling

Following institutional ethics committee review, ethical approval for the present study was obtained from the Institutional Review Board at the College of Medicine, King Saud University Medical City (IRB #—0117/2021). The present study was designed as a retrospective study evaluating the radiographic records of patients registered at the College of Dentistry and Dental University Hospital, King Saud University Medical City, Riyadh, Saudi Arabia.

For sample size estimation, based on previously reported evidence, a mean difference of 2.0 ± 3.19 mm in the measured linear values in OPG from that of CBCT images was considered as the expected mean significant difference. Accordingly, assuming statistical power greater than 80%, a confidence level of 95% and a significance level set at 0.05 (*p*-value < 0.05 indicates statistical significance), the minimum sample size was estimated to be 102 per group, for two equally matched comparison groups [25,26]. Therefore, a total sample size of 204 patient records having both OPG and CBCT digital radiographic data was targeted.

The samples were randomly selected from a pool of patient data available in the medical record database of the hospital, from those registered for dental treatment in the last 5 years. The inclusion of patient data in the selection pool was based on the following criteria [19]:

- Adult patients aged 18 years and older;
- Availability of digital OPG and CBCT images taken within an interval no longer than 7 days;
- No history of uncontrolled medical illnesses;
- No evidence of facial asymmetry, pathological lesions, fractures or surgeries in the mandible;
- Presence of at least 3/4 of dentition with bilateral mandibular molars (1st and 2nd), along with opposing maxillary molar teeth (to avoid variations in localization and measurement due to supra-eruption);
- No radiographic evidence of inordinate occlusal wear.

Selected patients were assigned a sequential numerical code, based on their chronological order of registration at the hospital. The final sample was selected with the help of an online random number generator (random.org), using a numerical code. Consent for the use of patient data without identifying information was obtained from the sample patients through a phone call.

### 2.2. Image Acquisition for OPG and CBCT

The quality of the acquired digital OPG and CBCT images was verified by an experienced oral radiologist, and, whenever the image quality was not satisfactory, the particular data were eliminated and a new patient was randomly included from the selection pool, in order to maintain the sample size. All OPG images were acquired using a digital panoramic radiography unit (Planmeca ProOne^®^, Helsinki, Finland) with an operational voltage of 120 kVp, maximum current of 2 mA and exposure time of 18 s. Similarly, all CBCT images were acquired using a digital CBCT unit (Planmeca Promax 3D Classic^®^, Helsinki, Finland), with an operational voltage and maximum current of 120 kVp and 5 mA, respectively. The CBCT exposure parameters included an “11 cm × 8 cm” field of view, a “0.3 mm × 0.3 mm × 0.3 mm” voxel resolution and a 0.1 mm slice thickness after image reconstruction.

As part of the standard operational protocol during OPG and CBCT image acquisition, patients were devoid of jewelry, hair accessories, eye glasses and removable dental appliances. Patients were asked to stand vertically erect, close to the imaging unit and holding its handles. After positioning the chin rest to align the occlusal table in a slight posteroinferior angulation, patients were asked to bite on the guiding stick with their anterior teeth and sagittal alignment was achieved by aligning the vertical laser marker between the eyes. During exposure, patients were instructed to remain stationary, swallow and close the lips and position their tongue towards the palate.

### 2.3. Radiographic Reference Points and Measurements

**Figure 2:**
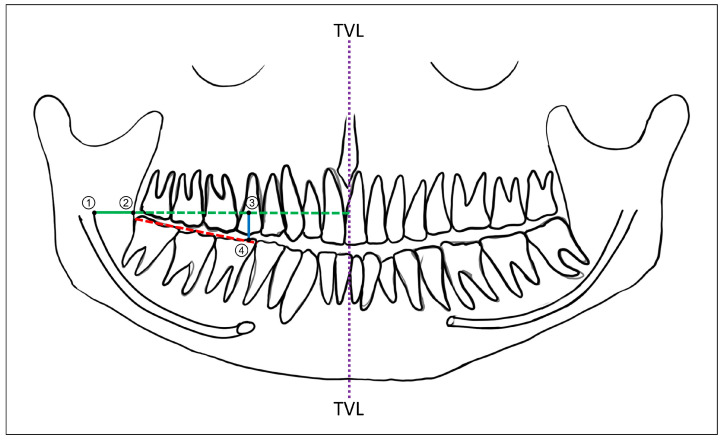
Representative line diagram of a panoramic radiographic image showing the markings for the measurement of the anteroposterior and superoinferior positions of the mandibular foramen (MF): TVL—true vertical line; green line—horizontal line extending perpendicular up to TVL from anteroinferior aspect of MF; red line—mandibular occlusal plane corresponding to cusps of mandibular molars; blue line—vertical line (parallel to TVL) extending from green line to occlusal plane (red line); point 1—MF; point 2—anterior border of ramus; linear distance from points 1 to 2—anteroposterior position of MF (in mm); and linear distance from points 3 to 4—superoinferior position of MF (in mm).

Radiographic reference points and linear digital measurements to localize the mandibular foramen using OPG and CBCT were performed on the digital radiographic images. All localizations and measurements on the radiographs were performed and recorded using the in-built linear scale tool of a radiographic image viewer software program (Planmeca Romexis^®^ Viewer Ver. 5.2.0.R, Helsinki, Finland). While digital OPG provided panoramic radiographic images as the output, for CBCT, the digitally reconstructed panoramic images using the curved planar reformation algorithm, based on the dental arch, were used [20]. The mandibular foramen was radiographically localized by determining its anteroposterior (AP) and superoinferior (SI) positions using three reference points. These points included the anterior border of the mandibular ramus (AB), the anteroinferior border of the mandibular foramen (MF) and the mandibular molar occlusal plane (OP). A horizontal line (MF-AB) perpendicular to the true vertical line (TVL) was drawn anteriorly from the MF until it reached the midline. The point where the MF-AB intersected the anterior border of the mandibular ramus was considered as the AB. Next, the occlusal plane (OPL) was determined by drawing a line connecting the cusps of the mandibular molar teeth, namely the 1st and 2nd molars. A vertical line, parallel to the true vertical, was drawn from the MF-AB to the OPL, at the level of the mesial cusp of the first mandibular molar, and the point where this line contacted the OPL was considered as the OP. Two linear measurements—namely MF to AB, indicating the AP position of the mandibular foramen, and MF-AB to OP, indicating the SI position of the mandibular foramen—were obtained digitally (Figure 2). Accordingly, the following quantitative outcome variables pertaining to the mandibular foramen position were recorded for comparison, based on independent variables (age, biological sex and anatomic localization) (Table 1).

### 2.4. Intra- and Inter-Examiner Reliability

**Figure 3:**
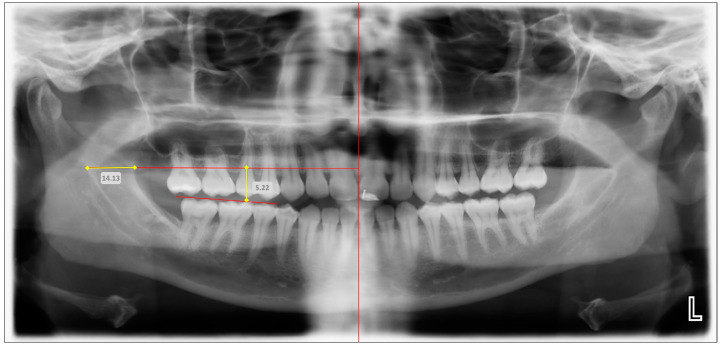
Representative image showing the digital measurements of the anteroposterior and superoinferior positions of the mandibular foramen (MF) (in mm) in an orthopantomogram (OPG). Red vertical line—true vertical line (TVL); Superior red horizontal line—perpendicular line drawn from anteroinferior aspect of MF to TVL; Inferior red horizontal line—mandibular occlusal plane; Horizontal yellow line—anteroposterior position of MF (in mm); and Vertical yellow line—superoinferior position of MF (in mm).

**Figure 4:**
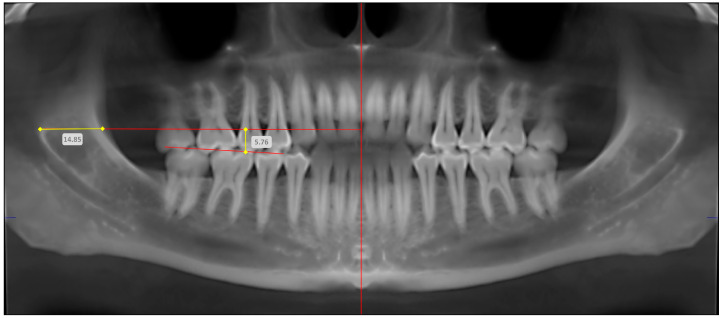
Representative image showing the digital measurements of the anteroposterior and superoinferior positions of the mandibular foramen (in mm) in a cone-beam computed tomography image (CBCT). Red vertical line—true vertical line (TVL); Superior red horizontal line—perpendicular line drawn from anteroinferior aspect of MF to TVL; Inferior red horizontal line—mandibular occlusal plane; Horizontal yellow line—anteroposterior position of MF (in mm); and Vertical yellow line—superoinferior position of MF (in mm).

Representative panoramic radiographic images obtained using OPG and CBCT, and the means of localizing the mandibular foramen using linear measurements, are depicted in Figure 3 (OPG) and Figure 4 (CBCT). Radiographic reference points and measurements were achieved by two independent examiners, who were trained and supervised by an oral radiologist. In order to ensure intra-examiner and inter-examiner reliability, the examiners were tested on a pilot sample of 15 radiographic records (OPG and CBCT). The examiners were further tested with the same pilot data after 10 days, and all aforementioned assessments were compared against data recorded by the oral radiologist, to validate the consistency and accuracy of the examinations. Moreover, repeated assessments by the examiners over time enabled the evaluation of potential intra-examiner variations.

### 2.5. Statistical Analysis

All statistical tests were performed using a statistical software package (IBM SPSS Statistics Version 21.0, IBM Corp., Armonk, NY, USA). Descriptive statistical analysis (frequencies and measures of central tendency and dispersion) were calculated for all independent and dependent variables. The Kolmogorov–Smirnov test was conducted to test the normality in the distribution of the quantitative variables. Paired *t*-tests were conducted to examine statistical differences between the individual measurements of the variables obtained using OPG and CBCT. In addition, the internal consistency of each measurement performed using OPG and CBCT was tested using reliability statistics (Cronbach’s alpha). Independent *t*-tests and one-way analysis of variance (ANOVA) with Tukey’s post hoc test were performed to compare the mean differences in the linear measurements (between OPG and CBCT) based on the age, biological sex and anatomic localization. Considering a 95% significance level, any *p*-value less than 0.05 indicated a statistically significant difference or association.

## 3. Results

Out of the 204 patient records examined in this study, the majority were females (*n* = 104; 50.9%). The age of the patients included ranged from 18 to 66 years, with a mean age of 34.65 years (SD—11.55; median—31 years). Stratifying the patients according to their age, the majority of the patients were in the age group of 26–40 years (*n* = 122; 59.8%), followed by those 41 years or older (*n* = 50; 24.5%) and those between 18 and 25 years of age (*n* = 32; 15.7%). The reliability of the linear digital measurements based on OPG and CBCT images was indicative of near-perfect agreement, as the Cohen’s kappa coefficient for intra- and inter-examiner reliability was 0.84 and 0.81, respectively. The test for the normality of the data showed a normal distribution for all measured quantitative variables (*p* < 0.05).

### 3.1. Accuracy of OPG in Determining Mandibular Foramen Location

In general, the mean values of the linear measurements used to locate the mandibular foramen using OPG were slightly lower than the values obtained using CBCT. However, the paired *t*-test comparison revealed no significant difference between OPG and CBCT in localizing the mandibular foramen in the anteroposterior aspect, on both the right and left sides of the mandible (MF-AP-OPG right—13.53 ± 2.44 mm; MF-AP-CBCT right—13.61 ± 2.39 mm/MF-AP-OPG left—13.19 ± 2.25 mm; MF-AP-CBCT left—13.36 ± 2.19 mm). Similarly, the superoinferior position of the mandibular foramen on the left side, as measured by OPG (MF-SI-OPG left—5.41 ± 1.65 mm), was not significantly different from that obtained with CBCT (MF-SI-CBCT left—5.52 ± 1.61 mm). In contrast, the OPG-based superoinferior localization of the right mandibular foramen (MF-SI-OPG right—5.25 ± 1.71 mm) was significantly different and lower than the mean linear measurement obtained through CBCT (MF-SI-CBCT right—5.59 ± 1.66 mm) (Table 2). Nevertheless, the mean difference between MF-SI-OPG and MF-SI-CBCT on the right side was minimal (0.34 ± 0.17; 95% C.I. 0.012–0.668; *p* < 0.05). The reliability tests for each measurement (MF-AP-right; MF-AP-left; MF-SI-right and MF-SI-left) obtained using OPG and CBCT indicated a high degree of internal consistency based on the calculated Cronbach’s alpha values (Table 2). The highest degree of internal consistency between the OPG- and CBCT-measured values was observed for the anteroposterior position of the right MF (0.949), followed by the superoinferior position of the right (0.939) and left (0.912) MF and lastly for the anteroposterior position of the left MF (0.903).

### 3.2. Comparison Based on Anatomic Location (Right or Left Side) and Demographic Variables (Age and Gender)

Comparing the right and left sides of the mandible, the differences between the OPG and CBCT measurements for the mandibular foramen were significantly different in both the anteroposterior (Table 3) and superoinferior aspects (Table 4). While, in terms of the anteroposterior position of the mandibular foramen, the difference was larger on the left side, it was greater for the right mandibular foramen in the superoinferior aspect. This indicates definitive differences in the accuracy of localizing the right and left mandibular foramen, owing to bilateral anatomic differences.

When the differences in the OPG and CBCT measurements were compared against the demographic variables (gender and age group), statistically significant differences were observed only for the anteroposterior position of the mandibular foramen between males and females (right side: t-statistic—4.125; *p*-value < 0.05/left side: t-statistic—4.839; *p*-value < 0.05) (Table 3). The aforementioned difference between the OPG and CBCT measurements was significantly smaller among females than in males, indicating a greater degree of anteroposterior anatomic variation in the mandibles of males. Although no statistically significant relationship could be established between the differences in the OPG and CBCT measurements and the patient age group, the smallest difference in terms of the anteroposterior position of the mandibular foramen was observed in patients aged 18 to 25 years (Table 3). A similar association could not be determined due to considerable variation with respect to the superoinferior position of the mandibular foramen, based on both the age group and gender (Table 4). A multivariate analysis to determine the effect of the statistical interaction between the gender and age group on the differences in the OPG and CBCT measurements revealed no significant relationship for the intercept model (F = 0.699; *p*-value = 0.696).

## 4. Discussion

Radiographic imaging techniques have become quintessential for clinical practice in dentistry and oral and maxillofacial surgery. Although several radiographic techniques are available, OPG and CBCT are among the most commonly used radiographs for diagnosis and treatment planning [1]. The present study was based on the premise that both the above radiographic techniques (OPG and CBCT) are equally accurate in locating the MF, and we attempted to prove this statistically. Accordingly, 204 patient records without any missing data were evaluated and the reliability of the examination was confirmed by a pilot study, which indicated near-perfect intra- and inter-examiner agreement.

In an effort to radiographically locate the MF on digital OPG and CBCT, linear measurements for the anteroposterior and superoinferior orientation were used. The measurements used in the present study, namely the distances from the MF to the AB and the MF-AB line to the OPL, were adapted from the study reported by Lasemi et al. [19]. In their study, based on panoramic radiographs, linear measurements were used to locate the MF as a guide to optimize the IAN block technique, and the researchers suggested a needle target site located approximately 5 mm superior to the occlusal plane and 16.5 mm behind the anterior border of the ramus [19]. Although the aforementioned study’s findings were similar to those of the present study in terms of the mean values [19], the accuracy of the OPG-based measurements was not correlated with any benchmark, unlike in the present study. Moreover, the present study used direct digital measurements obtained from the imaging software package (Planmeca Romexis^®^ Viewer Ver. 5.2.0.R, Helsinki, Finland), which was not only an advantage with digital OPG but also contributed to the accuracy and reproducibility. Alternatively, the tracing of conventional panoramic radiographs to locate the MF has also been reported. Trost et al. [16] used markings for the anterior and posterior border of the ramus and the mandibular notch to formulate a ratio, which helped to locate the MF during ramus osteotomies [16]. While the current study did not use any digital tracing techniques, the localization of the MF was based on similarly comparable landmarks and was consistently reproducible based on the outcomes reported. In addition, the present findings are coherent with those reported by Patil et al. [12] and Kaur et al. [14]. Both of these studies evaluated the accuracy of localizing the MF on OPGs by comparing them with measurements performed on dry mandibles [12,14]. Accordingly, OPGs were considered as a precise method of identifying the position of the MF, with their inherent magnification being the only factor for consideration [14]. However, in the present study, the issue of magnification was avoided by using linear measurement tools available in the digital image viewing software (Planmeca Romexis^®^ Viewer Ver. 5.2.0.R, Helsinki, Finland), which had inbuilt algorithms to provide precise measurements after adjustment for magnification [20].

In the present study, the statistical results showed no significant differences between the mean values recorded using digital OPG and CBCT for the anteroposterior position of the MF (Table 2). Although there was a statistically significant difference observed between the mean OPG and CBCT measurements for the superoinferior position of the MF on the right side alone (*p* = 0.042), this difference was minimal (0.34 ± 0.17 mm). Nevertheless, for the same measurement, the internal consistency between the values obtained though OPG and CBCT was remarkably high and less than only that of the anteroposterior position of the right MF (Table 2). In fact, for repeated measurements, similar to the one evaluated in the present study, the internal consistency is a reliable indicator of the accuracy [27]. In addition to the reliability of the outcomes reported, it is also suggestive of a reduction in errors during measurement and confidence in the interpreted values and forms a basis for further analysis [27]. Therefore, from a clinical standpoint, the above findings show the accuracy of the localization of the mandibular foramen using digital OPG to be similar to that of CBCT. Based on a study comparing OPG and CBCT for the analysis of anatomical variations in the mandible, Neves et al. [15] reported the better visualization and orientation of structures when using CBCT. However, they reported comparable outcomes with OPG and further stated its advantage as an easily available imaging modality, which could still be used to study mandibular anatomical variations [15]. In contrast, Mathew and Mohan [2] emphasized the accuracy of CBCT imaging in identifying mandibular anatomical structures and its importance before surgical procedures. Nevertheless, their study outcomes were based on techniques used to localize the mental foramen and evaluate the frequency of the anterior loop of the IAN, in a South Indian population [2]. In contrast, the present study focused on localizing the MF based on two linear measurements obtained from digital panoramic images.

Knowledge about the location of the MF and its anatomic variations is important for the success of IAN block anesthesia [10,28]. AlHindi et al. [29] reported a lack of knowledge about anatomy and inadequate training to overcome anatomic variations as the major causes of the failure of IAN blocks among dental students. In most clinical situations, the administration of an IAN block occurs at a target site slightly anterior to the actual position of the MF, thereby leading to a failure rate of up to 25% after the first LA injection [11]. This could be attributed to the absence of adequate guidance regarding the anteroposterior depth and superoinferior level of needle penetration. Studies based on CBCT suggest identifying the lingula and subsequently the MF as a means of improving the success rate of IAN block anesthesia [5]. While this is possible with 3D CBCT, panoramic radiographs only enable the two-dimensional visualization of the MF. This was the reason that the present study envisaged the localization of the MF based on two dimensions with reference to the anterior border of the ramus (for the anteroposterior position) and the mandibular occlusal plane (for the superoinferior position) [30]. Based on an assessment of the 3D reconstructed mandibular CT images of 260 patients, from all age groups (children/adolescents/adults), Feuerstein et al. [30] reported the mandibular occlusal plane and anterior border of the ramus as reliable reference points for the anatomical localization of the MF, irrespective of age differences [30]. According to their study, the location of the MF from the occlusal plane was 0.4–2.9 mm and that from the anterior border of the ramus was 17–19.5 mm [30]. These findings are slightly different from those of the present study, which could be attributed to the population demographics and variations arising as a result of the imaging technique and the study-based definition of the occlusal plane. Nevertheless, the aforementioned anatomic reference landmarks are not only clinically discernible, but their correlations with radiographic measurements indicate optimal accuracy when compared with CBCT [2,15]. Similar outcomes have been reported based on studies localizing the MF on dry mandibles, using both direct and conventional radiographic measurements [12,13,14].

Along with knowledge about the MF’s location, for successful IAN local anesthesia techniques, it is crucial to understand the anatomy of the MF and mandibular canal for orthognathic surgeries involving ramus osteotomies [31]. According to Trost et al. [16], the position of the MF based on conventional panoramic radiographs was in the anteroinferior two thirds of the mandibular ramus. They reported this as an arbitrary measure to guide oral and maxillofacial surgeons to plan their osteotomies in the mandibular ramus [16]. Similarly, Al-Shayyab [22] proposed a ‘40% rule’ based on CBCT to locate the MF, wherein any surgical intervention is to be avoided in the superior and posterior 40% of the lines connecting the mandibular notch to the inferior border and the anterior to posterior borders, respectively [22]. Although both of the above suggestions are based on anatomic reference points and landmarks, the authors not provide a definitive measurement, as reported in the present study. In another cadaveric study, Vorakulpipat et al. [3] evaluated the intraoperative concept of determining the anti-lingula position on the lateral surface of the ramus as a means of identifying the MF’s position on the medial surface [3]. Although the anti-lingula was identified in most mandibles with reliability and accuracy, its position rarely concurred with that of the MF on the medial surface ramus, thereby necessitating more accurate techniques for localization [3]. Considering the accuracy of anteroposterior and superoinferior measurements of the MF obtained with digital OPG in this study, it is therefore possible to hypothesize its use in achieving successful IAN local anesthesia and in planning ramus osteotomies.

In spite of the clinical relevance of digital OPG in identifying and accurately localizing the MF, as demonstrated in the present study, exceptional anatomical variations must be borne in mind. This is more commonly associated with mandibular edentulism, wherein a reduced ramus height, width and thickness lead to reduced anteroposterior and superoinferior measurements of the MF [32,33]. According to Prado et al. [32] and Matveeva et al. [33], measurements on dry mandibles indicated the MF to be closer to the anterior border of the ramus and almost at the same level as the residual alveolar ridge. Similarly, Shalini et al. [34] reported a 32.36% prevalence of accessory foramina in the vicinity of the MF, based on a study conducted on the dry mandibles of adults from a South Indian population. Based on a CBCT evaluation of pediatric dental patients, Vathariparambath et al. [35] reported, with reference to the occlusal plane, that the MF is located below it at 8 to 11 years of age, gradually shifting to the occlusal level by the age of 12 to 14 years, and it moves further posterosuperiorly by 15 to 18 years of age [35]. Owing to the specific objectives of the present study, the presence of anatomical variations was not considered as part of the methodology. However, when comparing OPG- and CBCT-based mean values in the Middle Eastern population, significant variation in the position of the MF between the right and left sides was observed. Similarly, the difference in the anteroposterior measurement of the MF between OPG and CBCT was significantly larger among males. It is therefore important that anatomical variations in the MF arising as a result of age, biological sex, edentulism and population subsets are given due clinical consideration during IAN blocks and mandibular ramus osteotomies.

Although the present study was able to determine, with considerable accuracy, the position of the MF from the anterior border of the ramus (right—13.53 ± 2.44 mm/left—13.19 ± 2.25 mm) and with reference to the mandibular occlusal plane (right—5.25 ± 1.71 mm/left—5.41 ± 1.65 mm), using digital OPG, there are certain limitations to be considered. Firstly, the study was based on a retrospective data set that had a specific inclusion criterion pertaining to dentate patients only. Secondly, the population studied was only from a single geographical region, with patients of similar anthropological characteristics. Lastly, the digital radiographic images compared in the study were analyzed using an imaging software package (Planmeca Romexis^®^ Viewer Ver. 5.2.0.R, Helsinki, Finland), the error ratios of which were not considered. Although the aforementioned limitations are capable of inducing bias when extrapolating the outcomes globally, their clinical relevance cannot be disregarded. Indeed, a strength of the present study is the fact that it is the first of its kind reported in the Middle Eastern population, where the availability of advanced CBCT imaging facilities is low in comparison to the more ubiquitous digital OPG. Moreover, the present study provides clinically discernible measurements to locate the MF based on visible and palpable anatomic landmarks, thereby helping dental, oral and maxillofacial surgeons in perfecting mandibular anesthesia techniques and orthognathic surgical planning [36]. Digital workflows and planning based on radiographs have enabled the accurate localization of the MF, more commonly with respect to mandibular orthognathic surgeries and, to some extent, for IAN anesthesia [6,17]. Nevertheless, the clinical relevance of the present study’s outcomes should be underlined, especially in training dental students and maxillofacial surgery residents. A pertinent example would be to encourage clinicians to conduct digital OPG-based anteroposterior and superoinferior measurements of the MF and to use this as guidance during the administration of IAN local anesthesia. The use of traditional anatomic reference points, along with measurements, would help to accurately identify the point of needle insertion and its height from the occlusal plane, as well as the depth of needle penetration and angulation needed to optimally reach the MF. The same principles could be applied during preoperative planning for ramus osteotomies to achieve favorable clinical outcomes.

## 5. Conclusions

Based on the present study, it can be concluded that digital OPG is an accurate modality to locate the MF based on its anteroposterior distance from the anterior border of the ramus and its superoinferior position with respect to the mandibular occlusal plane. These measurements would be clinically beneficial to dental and oral surgeons to achieve optimum IAN block anesthesia based on preoperative panoramic radiographs. Similarly, it would assist maxillofacial surgeons in planning ramus osteotomies without neurovascular complications during mandibular orthognathic surgeries. It should, however, be borne in mind that, in spite of the advanced digital radiographic techniques, anatomic variations are possible between the right and left sides, as well as due to age and gender. Future large-scale multicentric studies are needed for the similar validation of digital OPG to localize the MF in other population subsets. Moreover, the use of artificial intelligence in coordination with emerging radiographic technologies to simplify image acquisition and data synthesis is an area to be considered for future research.

## Figures and Tables

**Table 1:** Description of quantitative outcome variables pertaining to mandibular foramen position.

Variable	Description	Radiographic Technique	Abbreviation Based on Anatomic Localization	
			Right	Left
Anteroposterior position of mandibular foramen	Horizontal distance from anteroinferior border of mandibular foramen to anterior border of mandibular ramus	OPG	MF-AP-OPG (Right)	MF-AP-OPG (Left)
		CBCT	MF-AP-CBCT (Right)	MF-AP-CBCT (Left)
Superoinferior position of mandibular foramen	Vertical distance from anteroinferior border of mandibular foramen to mandibular occlusal plane (at level of mesial cusp of first molar)	OPG	MF-SI-OPG (Right)	MF-SI-OPG (Left)
		CBCT	MF-SI-CBCT (Right)	MF-SI-CBCT (Left)

**Table 2:** Mean values (in mm) for anteroposterior and superoinferior positions of mandibular foramen measured based on orthopantomogram (OPG) and cone-beam computed tomography (CBCT) (*n* = 204).

Mandibular Foramen Position	Anatomic Localization	Imaging Technique		t-Statistic	*p*-Value	Cronbach’s Alpha
		OPG	CBCT			
		Mean ± SD (Range)	Mean ± SD (Range)			
Anteroposterior position	Right	13.53 ± 2.44 (7.1–20.2)	13.61 ± 2.39 (7.8–19.6)	0.335	0.738	0.942
	Left	13.19 ± 2.25 (7.7–18.1)	13.36 ± 2.19 (8.1–18.8)	0.773	0.439	0.903
Superoinferior position	Right	5.25 ± 1.71 (4.2–6.2)	5.59 ± 1.66 (4.5–6.6)	2.038	**0.042**	0.939
	Left	5.41 ± 1.65 (4.1–6.3)	5.52 ± 1.61 (4.6–6.5)	0.682	0.496	0.912

OPG—orthopantomogram; CBCT—cone-beam computed tomography; SD—standard deviation; bold text indicates statistically significant *p*-value.

**Table 3:** Difference in measurements of anteroposterior position of mandibular foramen (in mm) between orthopantomogram (OPG) and cone-beam computed tomography (CBCT), compared against anatomic location (right versus left) and independent demographic variables.

Independent Demographic Variable		Difference in Anteroposterior Position of Right Mandibular Foramen		Difference in Anteroposterior Position of Left Mandibular Foramen		Comparison between Right and Left Sides
		Mean ± SD	*p*-Value	Mean ± SD	*p*-Value	*p*-Value
Overall (*n* = 204)		0.08 ± 0.24	-	0.17 ± 0.22	-	**<0.01**
Gender	Male (*n* = 100)	0.24 ± 1.13	**0.045**	0.46 ± 1.29	**0.029**	-
	Female (*n* = 104)	0.08 ± 1.12 *		0.03 ± 1.49 *		
Age group	18–25 years (*n* = 32)	0.15 ± 0.94 *	0.454	0.74 ± 2.10 *	0.055	-
	26–40 years (*n* = 122)	0.11 ± 1.08		0.02 ± 1.49		
	≥41 years (*n* = 50)	0.15 ± 1.36		0.57 ± 2.62 *		

* indicates mean value measured by orthopantomogram (OPG) greater than that measured by cone-beam computed tomography (CBCT); bold text indicates statistically significant *p*-value.

**Table 4:** Difference in measurements of superoinferior position of mandibular foramen (in mm) between orthopantomogram (OPG) and cone-beam computed tomography (CBCT), compared against anatomic location (right versus left) and independent demographic variables.

**Independent Demographic Variable**		**Difference in Anteroposterior Position of Right Mandibular Foramen**		**Difference in Anteroposterior Position of Left Mandibular Foramen**		**Comparison between Right and Left Sides**
		**Mean ± SD**	***p*-Value**	**Mean ± SD**	***p*-Value**	***p*-Value**
Overall (*n* = 204)		0.34 ± 0.17	-	0.11 ± 0.16	-	**<0.01**
Gender	Male (*n* = 100)	0.33 ± 1.02	0.942	0.19 ± 0.75	0.321	-
	Female (*n* = 104)	0.34 ± 0.94		0.06 ± 1.08		
Age group	18–25 years (*n* = 32)	0.44 ± 1.22	0.781	0.14 ± 0.81 *	0.157	-
	26–40 years (*n* = 122)	0.32 ± 0.96		0.21 ± 0.98		
	≥41 years (*n* = 50)	0.29 ± 0.87		0.08 ± 0.87		

* indicates mean value measured by orthopantomogram (OPG) greater than that measured by cone-beam computed tomography (CBCT); bold text indicates statistically significant *p*-value.

## Data Availability

The study datasets shall be made available upon justifiable request to the corresponding author.
